# Bioactive potential of green tea kombucha with propolis: *in vitro* bioavailability investigation

**DOI:** 10.3389/fnut.2026.1811711

**Published:** 2026-04-09

**Authors:** Şennur Ganimet, Seydi Yıkmış, Emad Karrar, Moneera O. Aljobair, Isam A. Mohamed Ahmed, Suleiman A. Althawab

**Affiliations:** 1 Department of Nutrition and Dietetics, Institute of Health Sciences, Namik Kemal University, Tekirdag, Türkiye; 2 Department of Food Technology, Tekirdag Namik Kemal University, Tekirdag, Türkiye; 3 Department of Plant Sciences, North Dakota State University, Fargo, ND, United States; 4 Department of Sports Health, College of Sports Sciences and Physical Activity, Princess Nourah bint Abdulrahman University, Riyadh, Saudi Arabia; 5 Department of Food Sciences and Nutrition, College of Food and Agricultural Sciences, King Saud University, Riyadh, Saudi Arabia

**Keywords:** bioavailability, green tea, kombucha tea, phenolic compound, propolis, response surface methodology

## Abstract

Kombucha tea, which is frequently preferred among functional drinks, is prepared by fermenting sweetened tea with a symbiotic colony of bacteria and yeast (SCOBY). Kombucha has various therapeutic potentials thanks to its rich bioactive components and high antioxidant capacity. Propolis, which has health benefits like antioxidant, antimicrobial and anti-tumor, can be added to improve the nutritional content of kombucha tea. The effectiveness of bioactive compounds in a beverage is linked to how well these compounds are absorbed by the body. Bioavailability refers to the portion of a dietary nutrient or bioactive compound that is usable for physiological processes and can be stored in the body. In this study, the *in vitro* bioavailability of green tea kombucha with propolis was investigated by adding propolis to improve kombucha tea's nutritional content. The study used the response surface methodology to obtain optimized green tea kombucha with propolis (GTK-P). Bioactive compound contents, bioavailability levels, and sensory analysis parameters of GTK-P samples and propolis-free kombucha (GTK) samples at 0, 7, 14, and 21 days were investigated comparatively. According to the results, bioactive compound content increased in both GTK and GTK-P samples as the storage period progressed. However, GTK-P had significantly higher bioactive compound concentrations and intestinal recovery rates (% recovery) relative to the GTK control (*p* < 0.05). In conclusion, GTK-P increases its therapeutic potential as a functional beverage with higher bioactive compound content and bioavailability. These findings reveal that kombucha with propolis could be a favorable functional food in terms of health-promoting effects.

## 1 Introduction

In Chinese medicine, kombucha—often called the “Tea of Immortality” or “Elixir of Life”— is traditionally prepared through the fermentation of sweetened tea using a symbiotic community of bacteria and yeast (SCOBY) (1, 2). During fermentation, many bioactive compounds in kombucha tea are dissolved into the liquid. Tea polyphenols significantly increase the antioxidant capacity of kombucha tea (3, 4). The health-protective mechanism of kombucha depends mainly on the metabolites produced by fermentation, the activity of polyphenols, and the combined effects of the tea's components (5). The substrate used, fermentation time, sugar concentration and SCOBY composition all have a bearing on the chemistry content (6). In summary, kombucha tea contains organic acids, sugars, alcohol, vitamins and minerals (7). Thanks to its antimicrobial (8), antioxidant (9), antidiabetic (10), anticancer (11) and hepatoprotective (12) effects, kombucha has important therapeutic potential.

Although kombucha can be made from various sources, including tea (13), vegetables (14) and fruit (15), traditional kombucha preparation uses green, black or oolong tea as a substrate (16). The green tea compound extracted from the *Camellia sinensis* plant is superior to black tea due to its positive health properties. Its main beneficial effects are attributed to its antioxidant capacity and polyphenol content (17). However, different substances can be added to kombucha to improve its nutritional content, such as its antioxidant and phenolic compound capacities (18, 19). One example of these substances is propolis (19). Propolis, commonly referred to as “bee glue,” is a natural resinous material produced by bees (including both honeybees and stingless bees) by mixing plant-derived substances with salivary enzymes and beeswax (20–22). The chemical composition of propolis may change depending on the bee species, the plant source used in propolis production, climatic conditions, the harvest season and the geographical location (20). Propolis has several beneficial health outcomes. These contain antioxidant, antimicrobial, anti-tumor, and anti-ulcer qualities. Propolis can also lower blood pressure, strengthen the immune system, and treat skin conditions (23, 24). Due to its therapeutic potential, propolis has recently attracted significant interest and is widely used in food and beverages, with claims that it can improve and protect human health (23).

Phenolic compounds' positive effects on people's health are related to their digestive properties, defined as bioavailability and bioaccessibility (25). Although bioavailability is expressed in various ways, it is generally defined as the fraction of an ingested nutrient or bioactive compound that is absorbed and available for physiological functions (26). Bioaccessibility, on the other hand, is referred to as the usable portion of the compound present in the food composition, which is released from the food matrix and ready to be absorbed in the gastrointestinal system (27). *In vitro* simulated gastrointestinal digestion can help determine the bioavailability of bioactive compounds (28). In addition, the lower cost and faster implementation of *in vitro* simulated bioaccessibility methods, the easier control of experimental variability compared to *in vivo* methods and the ethical considerations associated with *in vivo* studies all highlight *in vitro* digestion approaches as an alternative method to assess bioaccessibility (29, 30).

The high bioavailability of bioactive compounds in foods with positive health effects allows us to benefit more from their healing and protective properties. The main aim of our study is therefore to investigate the effectiveness of kombucha tea fermented with green tea and propolis, which is rich in bioactive compounds, in increasing bioavailability. Additionally, we plan to create a health-promoting product by optimizing the bioactive compound concentrations in the product obtained in our research.

## 2 Materials and methods

### 2.1 Preparation of kombucha teas

For the production of kombucha tea, green tea, granulated sugar, water-soluble propolis and kombucha mushroom obtained from the cultures under protection in Tekirdag Namik Kemal University Nutrition and Dietetics Laboratory were used. The kombucha tea was produced in the same laboratory. The produced kombucha teas were analyzed on days 0, 7, 14, and 21 of storage.

#### 2.1.1 Preparing kombucha tea fermented with green tea

It was labeled green tea fermented kombucha tea (GTK) and its production process was adapted based on the study by Cardoso et al. (31). To produce GTK, 100 g/L of sucrose and 12 g/L of green tea were added to boiled water and left to infuse for 15 min. The brewed tea was then filtered and left to cool to room temperature. Subsequently, pre-produced kombucha tea was added at 100 mL/L as the starter liquid, and SCOBY was incorporated at 10% (w/v). The mixture was incubated for 14 days in a dark environment at 28 ± 1 °C in an oven, with the vessel covered using a hygienic cotton cloth.

#### 2.1.2 Preparing kombucha tea with propolis fermented with green tea

Green tea, propolis added kombucha tea was altered based on the Bacanak and Keyvan (19) method. After 100 g/L of sugar was added to boiled water, green tea was put in and left to steep for 15 min. Pre-produced kombucha tea was then added at 100 mL/L as the starter liquid, together with SCOBY at 10% (w/v) and water-soluble propolis (Egriçayir, Türkiye). The jar was covered with a hygienic cotton cloth and incubated for 14 days in a dark environment at 28 ± 1 °C. Response surface methodology (RSM) was employed to determine the optimum green tea-to-propolis ratio during kombucha fermentation. The optimized propolis-enriched green tea kombucha sample was coded as GTK-P.

#### 2.2 Response surface methodology (RSM)

Minitab Statistical Analysis Software (version 18.1.1) was used to analyze RSM and investigate how bioactive compounds influence the preparation of kombucha tea enriched with propolis. Central Composite Design was selected as the experimental design and a 5-level, 2-factor experimental design was created. The independent variables were green tea (X1) and propolis (X_2_), while the dependent variables were total phenolic content (TPC), total flavonoid content (TFC), and total antioxidant capacity (DPPH).

The levels of the independent variables are expressed as follows: minimum value −1.41, center value 0, maximum value 1.41. Based on the experimental design in Table 1, 13 experiments were carried out using kombucha teas: green tea (X_1_), with a minimum of 6 g/L and a maximum of 14 g/L, and propolis (X_2_), with a minimum of 0.5% and a maximum of 2.00%.

**Table 1:** Optimisation of kombucha tea: levels of independent variables.

Independent variables		Levels				
		−1.41	−1	0	1	1.41
Green tea (g/L)	X_1_	6	8	10	12	14
Propolis (% vol)	X_2_	0.50	0.87	1.25	1.62	2.00

To find the relationship between independent variables (green tea and propolis) and the responses (TPC, TFC and DPPH), a second-degree polynomial formula (**Formula 1**) was used.


$$\begin{array}{l} y\;=\;\beta_{0}+\Sigma_{i=1}^{3}\beta_{i}X_{i}+\Sigma_{i=1}^{3}\beta_{ii}X_{i}^{2}+\Sigma_{\begin{array}{c} i=1\\ i<j \end{array}}^{3}\Sigma_{j=1}^{3}\beta_{ij}X_{i}X_{j} \end{array}$$ (1)

Formula definition: Dependent variable (y); independent variable (X_i_ and X_j_); intercept term (β_0_); coefficient of the first-order linear equation (β_i_); coefficient of the second-order linear equation (β_i*i*_); coefficient of the interaction between two factors (β_i*j*_).

### 2.3 Bioactive compounds

#### 2.3.1 Total phenolic compounds (TPC)

TPC was calculated using a modified Folin–Ciocalteu method (32). 0.1 mL of each of the prepared kombucha tea samples was taken and diluted with 4.5 mL of pure water. Then, 0.1 mL of Folin-Ciocalteu reagent was added to the sample and the mixture was vortexed for 1 min to homogenize it. The mixture was then left to stand for 3 min. Then, 0.3 mL of a 7.5% sodium carbonate solution was added, and the mixture was vortexed again. The samples were then left to incubate in the dark for 2 h. After this time, the absorbance of the samples was measured using a UV-VIS spectrophotometer (SP-UV/VIS-300SRB) at a wavelength of 765 nm, with a blank used for calibration. Results are shown in mg GAE/mg.

#### 2.3.2 Total flavonoid content (TFC)

TFC was analyzed using the approach explained by Zhishen et al. (33). For the TFC analysis, 1 mL of each sample was taken, to which 4 mL of pure water and 0.3 mL of 5% sodium nitrite solution were added. The mixture was then left to stand for 5 min. Then, 0.3 mL of a 10% aluminum chloride solution was added and the mixture was stirred. It was then left to stand for 6 min. Next, 2 mL of 1 M sodium hydroxide solution was added, followed by distilled water to bring the total volume up to 10 mL. Absorbance values were measured in a spectrophotometer at a wavelength of 510 nm, using a blank for calibration. Total flavonoids were expressed as milligrams of catechin equivalent per liter (mg CE/L).

#### 2.3.3 Antioxidant activity (DPPH)

To evaluate the antioxidant activity, the DPPH method (based on the inhibition of the DPPH (2,2-diphenyl-1-picrylhydrazyl) free radical developed by Brand-Williams et al. (34)) was used. Initially, 2.9 mL of a 0.1 mM DPPH solution prepared in ethanol was combined with 0.1 mL of kombucha tea and vortexed. The resulting mixture was incubated in the dark for 30 min before being measured using a spectrophotometer at a wavelength of 517 nm. DPPH radical scavenging activity was calculated according to **Formula 2**.


$$\begin{array}{l} DPPH\left(\%\right)=\left[\left(A_{0}-A_{1}\right)/A_{0}\right]10O \end{array}$$ (2)

A_0_: Absorbance of control / A_1_: Absorbance of kombucha tea.

### 2.4 Analysis of phenolic compounds

The polyphenol analysis of kombucha tea was determined using the procedure explained by Portu et al. (35), involving a chromatographic procedure with an ACE Generix C-18 column (250 × 4.6 mm, 5 μm packing, Agilent). Phenolic constituents were determined using an Agilent 1260 HPLC system fitted with a diode array detector (DAD). The mobile phase flow was adjusted to 0.80 mL/min, and the column temperature was kept constant at 30 °C. Gradient separation was performed using solvent A (water supplemented with 0.1% phosphoric acid) and solvent B (acetonitrile). The gradient started at 17% B and progressed in specified time intervals at specified percentages. In order: 15% (7 min), 20% (20 min), 24% (25 min), 30% (28 min), 40% (30 min), 50% (32 min), 70% (36 min) and 17% (40 min). Phenolic analysis was performed with an injection volume of 10 μL. As the analyses were performed in triplicate, the results for phenolic compounds were the average of three analyses (*n* = 3). The results were reported in micrograms per milliliter (μg/mL) of the mixture.

### 2.5 ICP-OES analysis of minerals

The mineral composition of kombucha tea (calcium (Ca), magnesium (Mg), iron (Fe), zinc (Zn), potassium (K) and manganese (Mn)) was determined by inductively coupled plasma optical emission spectroscopy (ICP-OES) analysis. Dissolution was performed using a Berghof Instruments Speedwave microwave digestion system (Berghof, Germany) (36). Mineral concentrations are expressed in milligrams per liter (mg/L).

### 2.6 *In vitro*-simulated gastrointestinal digestion analysis

The *in vitro* digestion process of kombucha tea examples was carried out using the protocol developed by Minekus et al. (37). Briefly, the samples were first subjected to the oral phase by mixing with simulated salivary fluid containing α-amylase at pH 7.0 and incubating at 37 °C under constant shaking. Subsequently, the gastric phase was initiated by adjusting the pH to 3.0 and adding pepsin-containing simulated gastric fluid, followed by incubation at 37 °C under continuous shaking. Thereafter, the intestinal phase was performed by readjusting the pH to 7.0 and adding simulated intestinal fluid containing pancreatin and freshly prepared bile, and the samples were further incubated at 37 °C for intestinal digestion. At the end of each digestion phase, the samples were centrifuged (15 min, 4 °C, 7,340 rpm), and the supernatant was collected as the bioaccessible fraction for subsequent TPC and TFC analyses, while the undigested residue was discarded. Bioactive compounds (TPC and TFC) were analyzed after the gastric and intestinal stages had been followed. All digestion experiments were conducted in triplicate for each treatment group.

### 2.7 Sensory evaluation

Sensory evaluation of kombucha tea samples was performed using the protocol developed by Yıkmış et al. (38), with some modifications. Fifteen semi-trained panelists from Tekirdag Namik Kemal University, who had received prior training in sensory evaluation techniques, participated in the evaluation. The samples were evaluated based on their color, taste, odor, sourness, and overall palatability. To ensure accurate sensory perception, the panelists were given water to clean their palates and rinse their mouths between tasting sessions. A 9-point hedonic scale ranging from 0 to 9 was used to evaluate the sensory characteristics.

### 2.8 Statistical analysis

Data were analyzed using SPSS 22.0 software (SPSS Inc., Chicago, IL, USA). Three-dimensional response surface methodology (RSM) plots were produced with SigmaPlot 12.0 (Systat Software Inc., San Jose, CA, USA), and RSM calculations were performed using Minitab 18.1.1. The experimental results are expressed as the mean ± standard deviation (SD) of three replicates for each treatment. These were compared using the “one-way ANOVA-Tukey test” and the “independent samples *t*-test”. The significance level was determined as *p* < 0.05.

## 3 Result and discussion

### 3.1 Bioactive compound optimization

RSM has been used to find the best recipe for producing the best green tea kombucha with added propolis (GTK-P) by determining the optimal recipe for the dependent variables (TPC, TFC, and DPPH). Similarly, Reyes-Flores et al. (18) used RSM to optimize black tea kombucha to which hemp seeds were added to boost the antioxidant capacity and total phenolic content. The results of the optimization showed the second-order modeling equations for kombucha tea: TPC in **Formula 3**, TFC in **Formula 4**, and DPPH in **Formula 5**. According to these equations, an increase in the amount of green tea (X_1_), one of the independent variables, was found to have a positive linear effect on the TPC, TFC, and DPPH values. An increase in the amount of propolis (X_2_), another independent variable, was found to negatively affect the TPC, TFC and DPPH values. Additionally, the TPC, TFC, and DPPH values were negatively affected by the squared effects of the independent variables, but positively affected by their binary interactions.


$$\begin{array}{l} \text{TPC}\left(\text{mg GAE}/\text{L}\right)=230.68\;+\;15.516\;X_{1}-\;8.99\;X_{2}\\ -\;1.1116X_{1}X_{1}\;-\;22.80X_{2}X_{2}+\;7.700X_{1}X_{2} \end{array}$$ (3)

$$\begin{array}{l} \text{TFC}\left(\text{mg CE}/\text{L}\right)=33.28\;+\;2.167\;X_{1}\;-\;1.497X_{2}\\ -\;0.15614\;X_{1}X_{1}\;-\;3.152X_{2}X_{2}+\;1.0967X_{1}X_{2} \end{array}$$ (4)

$$\begin{array}{l} \text{DPPH}\left(\%\;\text{inhibition}\right)=84.18\;+\;1.360\;X_{1}\;-\;16.17X_{2}\\ -\;0.3220X_{1}X_{1}-\;11.612\;X_{2}X_{2}+\;4.197X_{1}X_{2} \end{array}$$ (5)

The TPC, TFC and DPPH experimental results and RSM analysis for kombucha tea with varying levels of green tea and propolis are given in Table 2. The TPC and TFC experimental analysis data were found to vary between 295.15 and 331.72 mg GAE/L and between 42.16 and 47.38 mg CE/L, respectively. The lowest TPC and TFC values were observed in the 13th trial, while the maximum values were observed in the 6th trial. The TPC and TFC results analyzed using RSM varied between 294.65 and 332.18 mg GAE/L and between 42.09 and 47.44 mg CE/L, respectively. The lowest and highest TPC and TFC values obtained as a result of RSM were observed in the 13th and 6th trials, respectively. The findings of the experiments demonstrated that the rate of DPPH inhibition ranged from 70.63% to 81.06%. The lowest value was found in the 12th experiment, while the highest value was observed in the 10th experiment. The lowest DPPH inhibition percentage obtained by RSM was 70.73% in the 12th trial, and the highest was 80.78% in the 10th trial. As shown in Table 2, the differences between the experimental and RSM data for the independent variables are very low. For example, the difference between the experimental TPC value of 303.27 mg GAE/L and the RSM value of 302.99 mg GAE/L in the second trial is only 0.09%. This demonstrates the high accuracy of the RSM model in predicting bioactive compounds (39).

**Table 2:** Experimental and RSM data for dependent variables.

Run No.	Independent variables		Dependent variables					
	Green Tea (X_1_)	Propolis (X_2_)	TPC (mg GAE/L)		TFC (mg CE/L)		DPPH (% inhibition)	
			Experimental data	RSM predicted	Experimental data	RSM predicted	Experimental data	RSM predicted
1	14.00	1.25	318.04	317.92	45.43	45.41	74.96	75.20
2	10.00	0.50	303.27	302.99	43.32	43.28	75.39	75.58
3	10.00	1.25	324.80	324.07	46.33	46.25	79.55	79.69
4	8.00	1.63	308.14	308.84	44.02	44.11	72.26	71.97
5	10.00	1.25	324.45	324.07	46.35	46.25	79.85	79.69
6	12.00	1.63	331.72	332.18	47.38	47.44	79.41	79.02
7	12.00	0.88	311.96	312.55	44.56	44.62	75.62	75.23
8	10.00	1.25	324.20	324.07	46.12	46.25	79.48	79.69
9	10.00	1.25	324.58	324.07	46.37	46.25	79.87	79.69
10	8.00	0.88	311.48	312.31	44.49	44.59	81.06	80.78
11	10.00	1.25	323.57	324.07	46.26	46.25	79.12	79.69
12	10.00	2.00	319.84	319.50	45.72	45.67	70.63	70.73
13	6.00	1.25	295.15	294.65	42.16	42.09	73.82	73.87
GTK-P	12.34	1.57	331.76		47.38		79.24	
Experimental values			338.84		44.51		77.45	
% Difference			2.1%		6.1%		2.3%	

TPC, total phenolic compound; TFC, total flavonoid content; DPPH, 1,1-Diphenyl-2-picrylhydrazyl; GAE, gallic acid equivalent; CE, catechin equivalent; RSM, Response surface methodology; GTK-P, Kombucha tea fermented with green tea with optimized propolis addition.

Table 2 shows the maximum optimisation values for producing kombucha tea fermented with green tea and propolis, which contain optimal levels of bioactive compounds. According to these values, the amount of green tea for GTK-P was found to be 12.34 g/L, while the amount of propolis was found to be 1.57%. Based on these ratios, the RSM values for TPC, TFC and DPPH inhibition were found to be 331.76 mg GAE/L, 47.38 mg CE/L, and 79.24%, respectively. The TPC, TFC and DPPH values of the GTK-P samples obtained experimentally (0 days) were found to be 338.84 mg GAE/L, 44.51 mg CE/L, and 77.45% inhibition, respectively. Comparing the RSM-predicted results for bioactive compounds with the experimental results revealed that the TFC and DPPH values were 6.1% and 2.3% higher, respectively, while the TPC value was 2.1% lower. The results for the bioactive compounds were similar in the RSM and experimental analyses.

**Figure 1:**
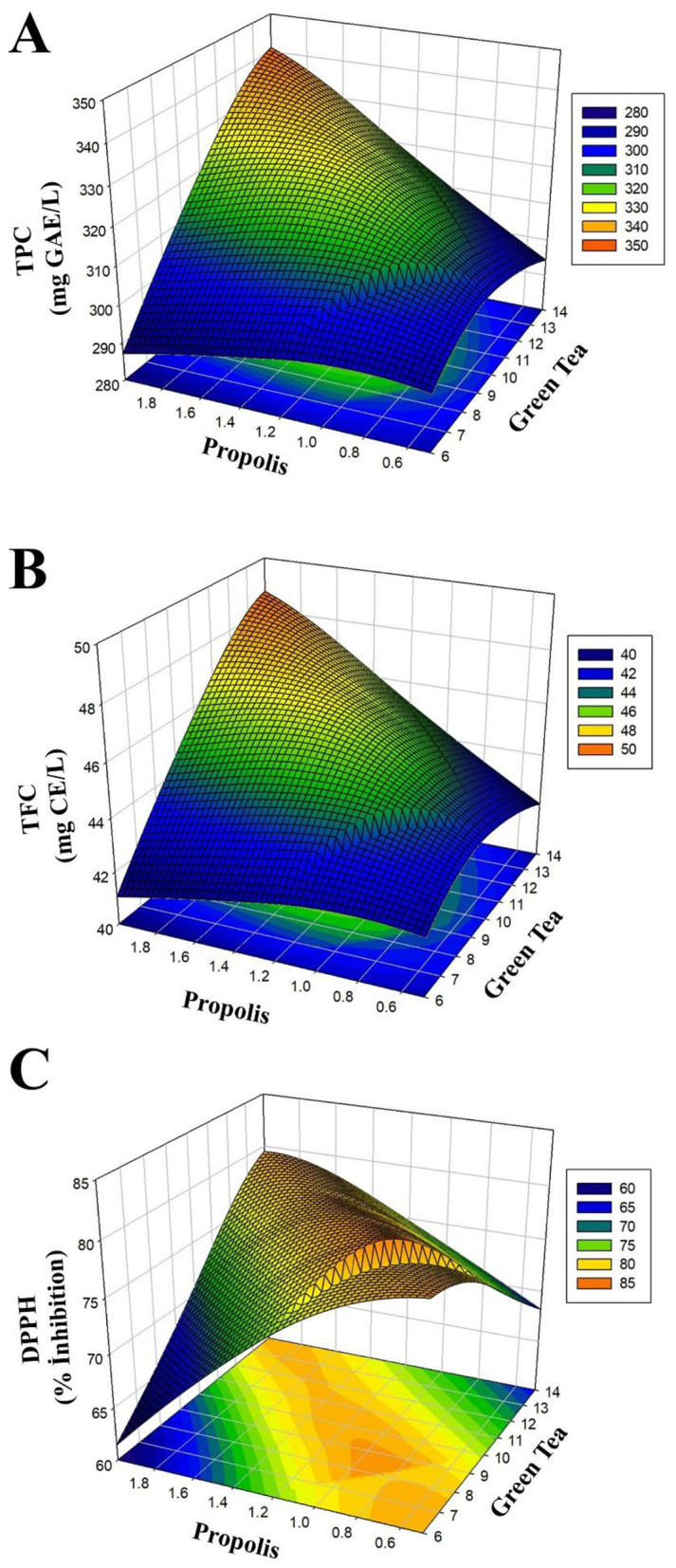
RSM graph showing the effect of the independent variables on TPC **(A)**, TFC **(B)** and DPPH **(C)**.

Figures 1A–C shows the surface response graphs explaining the influence of green tea and propolis amounts on TPC, TFC, and DPPH. As can be seen in these graphs, a rise in the amounts of green tea and propolis has a positive impact on TPC, TFC, and DPPH values.

The analysis of variance (ANOVA) table for the total phenolic and flavonoid content and antioxidant capacity of kombucha tea, based on RSM results, is shown in Table 3. The F-values for TPC and TFC were found to be 560.97 and 462.20, respectively, with *p*-values of 0.000 for both. The TPC and TFC results for kombucha tea were significantly affected by the linear effects of green tea and propolis content, as well as by their two-way and square interactions (*p* < 0.01). The R^2^ values for TPC and TFC were 99.75% and 99.70%, respectively, indicating high predictive accuracy. The F-value of the DPPH inhibition model is 190.84 and the *p*-value is 0.000, indicating that the model is significant for DPPH inhibition. The *p*-values for the linear interactions of green tea (X1) and propolis (X_2_) are 0.019 and 0.000, respectively. This suggests that the amount of green tea has a considerable impact on the DPPH result (*p* < 0.05), whereas the amount of propolis has an even more significant effect (*p* < 0.01). The DPPH results for kombucha tea are statistically significantly affected by two-way and square interactions of the amounts of green tea and propolis (*p* < 0.01).

**Table 3:** ANOVA results for the TPC, TFC, and DPPH content of kombucha tea, as obtained with RSM.

Source	DF	TPC (mg GAE/L)		TFC (mg CE/L)		DPPH (% inhibition)	
		F-value	*P*-value	F-value	*P*-value	F-value	*P*-value
Model	5	560.97	0.000	462.20	0.000	190.84	0.000
Linear	2	663.53	0.000	558.53	0.000	66.06	0.000
X_1_	1	882.61	0.000	737.66	0.000	9.29	0.019
X_2_	1	444.45	0.000	379.39	0.000	122.83	0.000
Square	2	594.05	0.000	476.48	0.000	272.60	0.000
X_1_X_1_	1	983.63	0.000	795.96	0.000	265.58	0.000
X_2_X_2_	1	511.49	0.000	401.01	0.000	426.88	0.000
2-Way Interaction	1	289.67	0.000	240.98	0.000	276.89	0.000
X_1_X_2_	1	289.67	0.000	240.98	0.000	276.89	0.000
Error	7						
Lack-of-Fit	3	3.49	0.129	1.22	0.412	2.19	0.232
Pure Error	4						
Total	12						
R^2^		99.75%		99.70%		99.27%	
Adj. R^2^		99.57%		99.48%		98.75%	
Pred. R^2^		98.61%		98.73%		96.36%	

X_1_, Green tea; X_2_, propolis; TPC, total phenolic compound; TFC, total flavonoid content; DPPH, 1,1-Diphenyl-2-picrylhydrazyl; GAE, gallic acid equivalent; CE, catechin equivalent; DF, degrees of freedom; R^2^, coefficient of determination; *p* < 0.05, significant differences; *p* < 0.01, very significant differences.

Results: The differences between the experimental and RSM data for bioactive compounds are very low. This demonstrates that the surface response method provides consistent and accurate results for bioactive compounds. According to the results of the ANOVA analysis, the model used for bioactive compounds is significant.

### 3.2 Bioactive components

The TPC, TFC, and DPPH results for the GTK and GTK-P samples, categorized by storage day, are presented in Table 4. The total phenolic content of the GTK samples showed a slight increase during storage. Similar TPC levels for green tea kombucha have been reported in the literature. For instance, Jakubczyk et al. (40) and Fraiz et al. (41) found TPC values of 320 mg GAE/L for green tea kombucha, close to our results. Conversely, Cardoso et al. (31) reported a TPC value of 0.70 ± 0.09 mg GAE/mL for green tea kombucha at the conclusion of the 10-day fermentation process, revealing a higher phenolic content than in our study. The TPC value of the GTK-P sample was found to be 337.05 ± 3.01 mg GAE/L after 14 days of fermentation (day 0). In a research by Bacanak and Keyvan (19), kombucha samples fermented with black tea and propolis added at different ratios (0.5–2.0%) exhibited TPC values of 1,240.98 ± 0.00, 1,376.43 ± 0.00, 1,941.02 ± 0.00, and 1,726.02 ± 0.00 mg GAE/L by day 14 of fermentation. When compared with the data obtained from our study, these results suggest that black tea kombucha contains higher levels of total phenolic compounds than green tea kombucha. This may be related to the type of tea and the amount of propolis used.

**Table 4:** TPC, TFC, and DPPH results of GTK and GTK-P samples.

Storage (Days)	TPC (mg GAE/L)		TFC (mg CE/L)		DPPH (% inhibition)	
	GTK	GTK-P	GTK	GTK-P	GTK	GTK-P
Day 0	310.31 ± 1.66aA	337.05 ± 3.01aB	38.78 ± 0.70aA	46.13 ± 1.46aB	68.17 ± 1.67aA	77.47 ± 1.90aB
Day 7	313.34 ± 1.51abA	341.20 ± 2.61abB	38.91 ± 0.70aA	44.95 ± 1.00aB	68.99 ± 1.69aA	78.40 ± 1.92aB
Day 14	315.85 ± 1.03bcA	344.12 ± 2.60bcB	39.37 ± 1.03aA	46.38 ± 0.50aB	70.11 ± 2.14aA	79.22 ± 1.77aB
Day 21	317.95 ± 1.82cA	348.49 ± 1.55cB	39.95 ± 0.72aA	45.51 ± 2.07aB	70.42 ± 2.50aA	79.95 ± 1.66aA

TPC, total phenolic compound; TFC, total flavonoid content; DPPH, 1,1-Diphenyl-2-picrylhydrazyl; GAE, gallic acid equivalent; CE, catechin equivalent; GTK, Kombucha tea fermented with green tea; GTK-P, Kombucha tea fermented with green tea with optimized propolis addition. The results are presented as the mean ± standard deviation. Different lowercase letters (a–c) within the same column indicate statistically significant differences between storage days (*p* < 0.05). Different uppercase letters (A–B) within the same row indicate statistically significant differences between products on the same day (*p* < 0.05).

TPC and TFC results increased steadily in the GTK samples over the 21-day storage period. While an increase in TPC was observed in the GTK-P sample, TFC levels showed minor fluctuations during storage (Table 4). The TPC value in GTK samples increased by 2.4% from day 0 to day 21; in GTK-P samples, this increase was 3.3%, higher than in GTK samples. Adding propolis to kombucha tea resulted in a greater increase in TPC values during storage. Similarly, adding propolis to black tea-based kombucha was found to increase TPC levels by between 18% and 48% (19). In a study by Lopes et al. (42), adding propolis to red fruit juice resulted in minimum and maximum increases in TPC values of 41.8% and 91.2%, respectively, compared to the control group (fruit juice without propolis). Additionally, flavonoid content, which was not detected in the control group, was detected at levels of 14–22 mg QE/100 g when propolis was added. The TPC and TFC results of the GTK-P samples on days 0, 7, 14, and 21 were statistically higher than the corresponding GTK values at all time points (*p* < 0.05).

The DPPH radical scavenging activity of GTP samples was observed to be between 68% and 70% over different storage periods. Silva et al. (43) and Jakubczyk et al. (40) reported higher DPPH radical scavenging capacities for green tea kombucha at the end of 14 days of fermentation than were observed in this study, with inhibition rates of 92.5 ± 1.1% and 88.23 ± 0.83%, respectively. Total antioxidant capacity increased slightly in both the GTK and GTK-P sample groups over a 21-day storage period. This increase was similar for both GTK (3.2%) and GTK-P (3.1%) samples. A study investigating the effect of storage on antioxidant capacity observed a 7.4% decrease in DPPH values in black tea kombucha over 30 days (44). The DPPH results for the GTK-P samples (on days 0, 7, 14, and 21) were consistently higher than the corresponding GTK values. According to Lopes et al. (42), total antioxidant capacity increased significantly with increasing propolis doses (3.1, 4.6, and 6.1 mg/mL). Propolis has the potential to prolong the storage life of food products and improve their overall quality and stability (45).

### 3.3 Phenolic components

The phenolic compound content of GTK and GTK-P samples on different storage days is presented in Table 5. Eight different phenolic compounds were detected in the GTK samples. The most abundant compound was catechin hydrate (164.58 ± 3.44 μg/mL), and the least abundant compound was t-ferulic acid (0.12 ± 0.00 μg/mL). In the GTK-P samples, the phenolic profile also included chrysin, salicylic acid and rosmarinic acid, in addition to these compounds. Phenolic compounds such as chrysin and rosmarinic acid are reported to be the main components of the characteristic propolis phenolic profile, contributing to its antioxidant potential and biological activities (46, 47). Notably, the persistence of the chrysin compound in the GTK-P samples despite its decrease from 63.16 μg/mL to 18.24 μg/mL over time suggests the effective integration of propolis-derived flavonoids into the fermentation matrix. These findings reveal that the addition of propolis enriches the phenolic profile of the product. However, the gradual decrease in chrysin concentration during storage may be related to various factors affecting flavonoid stability. Flavonoids such as chrysin are susceptible to degradation due to environmental factors, including oxidizing agents, water, light and changes in pH (48). Furthermore, flavonoids may interact with metal ions or other matrix components, which can also affect their stability (49, 50). Despite this decline, measurable levels of chrysin persist.

**Table 5:** Phenolic compounds and minerals result of GTK and GTK-P samples.

Studied Compound		Samples	Storage (Days)			
			Day 0	Day 7	Day 14	Day 21
Phenolic compounds (μg/mL)	Chlorogenic acid	GTK	4.33 ± 0.12bA	6.24 ± 0.13dA	3.42 ± 0.05aA	5.68 ± 0.08cA
		GTK-P	4.44 ± 0.06aA	4.24 ± 0.06aB	4.16 ± 0.14aA	n.d
	Catechin hydrate	GTK	162.34 ± 4.50aA	164.58 ± 3.44aA	162.98 ± 2.33aA	164.53 ± 2.30aA
		GTK-P	178.56 ± 2.55bA	188.20 ± 2.69bB	160.55 ± 5.54aA	186.92 ± 2.62bB
	Caffeic acid	GTK	2.84 ± 0.08aA	2.65 ± 0.06aA	10.99 ± 0.16cA	9.17 ± 0.13bA
		GTK-P	11.39 ± 0.16cB	2.80 ± 0.04aA	10.49 ± 0.36bA	12.86 ± 0.18dB
	p-Coumaric acid	GTK	n.d	n.d	n.d	n.d
		GTK-P	n.d	n.d	n.d	n.d
	Rutin	GTK	29.26 ± 0.81cA	26.08 ± 0.55bA	26.70 ± 0.38bA	23.50 ± 0.33aA
		GTK-P	40.05 ± 0.57bB	35.91 ± 0.26aB	33.45 ± 1.15aA	33.49 ± 0.47aB
	t-Ferulic acid	GTK	0.46 ± 0.01dA	0.16 ± 0.00bA	0.12 ± 0.00aA	0.42 ± 0.01cA
		GTK-P	1.54 ± 0.02cB	0.95 ± 0.01aB	0.90 ± 0.03aB	1.08 ± 0.02bB
	Naringin	GTK	n.d	n.d	n.d	n.d
		GTK-P	n.d	n.d	n.d	n.d
	o-Coumaric acid	GTK	0.34 ± 0.01aA	0.49 ± 0.01cA	0.37 ± 0.01bA	0.53 ± 0.01dA
		GTK-P	0.52 ± 0.01bB	0.40 ± 0.01aB	0.49 ± 0.02bB	0.50 ± 0.01bA
	Rosmarinic acid	GTK	n.d	n.d	n.d	n.d
		GTK-P	n.d	1.09 ± 0.02A	n.d	n.d
	Salicylic acid	GTK	n.d	n.d	n.d	n.d
		GTK-P	0.02 ± 0.00aA	1.65 ± 0.02dA	0.42 ± 0.01bA	0.54 ± 0.01cA
	Resveratrol	GTK	1.13 ± 0.03bA	0.45 ± 0.01aA	0.45 ± 0.01aA	0.41 ± 0.01aA
		GTK-P	0.48 ± 0.01bB	0.52 ± 0.01bB	0.43 ± 0.01aA	0.57 ± 0.01cB
	Quercetin	GTK	n.d	1.04 ± 0.02aA	0.99 ± 0.01aA	1.22 ± 0.02bA
		GTK-P	1.53 ± 0.02bA	1.36 ± 0.02aB	1.64 ± 0.06bB	n.d
	Chrysin	GTK	n.d	n.d	n.d	n.d
		GTK-P	63.16 ± 0.9dA	48.88 ± 0.34cA	34.60 ± 1.19bA	18.24 ± 0.26aA
	Total	GTK	200.68 ± 5.56aA	201.69 ± 4.22aA	206.01 ± 2.94aA	205.46 ± 2.88aA
		GTK-P	301.70 ± 4.31bB	286.00 ± 2.79bB	247.13 ± 8.52aA	254.21 ± 3.56aB
Minerals (mg/L)	Ca	GTK	8.84 ± 0.13aA	8.66 ± 0.12aA	9.34 ± 0.13bA	9.90 ± 0.07cA
	GTK-P	13.68 ± 0.29aB	16.46 ± 0.23bB	17.83 ± 0.25cB	12.66 ± 0.26aB
	Mg	GTK	5.44 ± 0.04bA	5.63 ± 0.04cA	5.76 ± 0.04cA	3.62 ± 0.03aA
		GTK-P	7.40 ± 0.15bB	7.25 ± 0.10bB	7.55 ± 0.11bB	6.13 ± 0.17aB
	Fe	GTK	1.06 ± 0.02cA	0.84 ± 0.01bA	0.86 ± 0.01bA	0.63 ± 0.00aA
		GTK-P	1.36 ± 0.03cB	1.21 ± 0.02bB	0.78 ± 0.01aB	1.28 ± 0.03bcB
	Zn	GTK	0.49 ± 0.01cA	0.44 ± 0.01bA	0.49 ± 0.00cA	0.32 ± 0.00aA
		GTK-P	0.69 ± 0.01dB	0.62 ± 0.01cB	0.51 ± 0.01bA	0.33 ± 0.01aA
	K	GTK	201.11 ± 2.87dA	177.96 ± 2.54cA	155.02 ± 1.10bA	129.15 ± 0.92aA
		GTK-P	263.25 ± 5.50bB	248.46 ± 3.55bB	185.31 ± 2.59aB	172.72 ± 3.61aB
	Mn	GTK	13.48 ± 0.29bA	21.06 ± 0.15dA	18.43 ± 0.13cA	12.24 ± 0.09aA
		GTK-P	28.52 ± 0.60dB	25.92 ± 0.37cB	18.59 ± 0.26aA	23.74 ± 0.50bB

GTK, Kombucha tea fermented with green tea; GTK-P, Kombucha tea fermented with green tea with optimized propolis addition. The results are presented as the mean ± standard deviation. Different lowercase letters (a–c) within the same row indicate statistically significant differences between storage days (*p* < 0.05). Different uppercase letters (A–B) within the same column indicate statistically significant differences between products on the same day (*p* < 0.05).

When evaluated in terms of total phenolic content, it was found that the level of total phenolics remained relatively constant (200.68–206.01 μg/mL) in GTK samples throughout the storage period, while GTK-P examples were found to have a significantly higher total phenolic content (301.70 μg/mL) at the outset (*p* < 0.05). One study examined the phenolic composition of various plants added to green tea and reported that hibiscus-added green tea kombucha had the highest total polyphenol content, while kombucha containing only green tea had a higher total polyphenol content than products with mint or *Clitoria ternatea* additions (51). Although a reduction in total phenolic content was observed as the storage period progressed, GTK-P samples consistently exhibited a higher total phenolic level than GTK samples. This suggests that propolis expands the phenolic pool and contributes to preserving phenolic compounds during fermentation and storage by enhancing the system's antioxidant potential. Indeed, the literature reports that propolis contributes to the preservation of antioxidant properties in food systems such as fruit and fruit juice, supporting the physical and chemical properties of food and helping to maintain product quality throughout the storage period (52).

Examination of Tables 4, 5 shows that the TPC value increased, whereas the total phenolic composition of the GTK-P samples decreased during the storage period. This apparent inconsistency may be attributed to differences in the analytical methods used. The Folin–Ciocalteu method, which is used to determine TPC, is not entirely specific to phenolic compounds, as it can also react with other reducing substances (53). Additionally, individual phenolic compounds may undergo degradation, oxidation, or enzymatic transformation during storage, which can alter the phenolic profile of the samples (54). Therefore, while changes occur in the phenolic profile, the TPC value may increase due to the presence of other reducing compounds.

### 3.4 Mineral compounds

Minerals are one of the inorganic substances that perform important functions in the human body. They are essential in small amounts for growth, maintenance, and the continuation of normal physiological functions (55). Table 5 presents the mineral content of GTK and GTK-P samples according to storage time. Six different minerals (Ca, Mg, Fe, Zn, K, and Mn) were detected in the kombucha tea samples. The mineral with the highest concentration in both sample groups was determined to be potassium (K), while zinc (Zn) was found to be the mineral with the lowest concentration. A study by Kluz et al. (56) also reported that potassium was the predominant mineral in kombucha tea.

Although potassium was the predominant mineral in both groups, GTK-P samples exhibited a richer electrolyte profile than GTK samples, reaching an initial level of 263.25 mg/L (*p* < 0.05). Analysis results revealed that adding propolis significantly enriched the mineral content of the kombucha matrix. In particular, the levels of Ca, Mg, Fe, Zn, K, and Mn were detected to be higher in the GTK-P examples than in the GTK examples at all storage points. While some minerals decreased with storage time, GTK-P samples largely maintained their superior mineral content, suggesting that propolis increases kombucha's functional value in terms of both bioactive compounds and mineral nutrition. This suggests that kombucha tea enriched with propolis may be a functional beverage with high mineral bioavailability. Literature data also support these findings. For example, Rendueles et al. (57) reported that calcium and potassium were the main minerals in the 31 different propolis samples they examined. Similarly, Izol and Turhan (58) reported that propolis has a rich mineral composition, primarily of potassium, iron, and magnesium.

### 3.5 *In vitro* bioaccessibility

The effects of the bioactive compounds (TPC and TFC) in the GTK and GTK-P samples on the *in vitro* gastrointestinal digestion model are presented in Table 6. The stability of these compounds can be affected by several factors, such as cell wall structure, the position of glycosides, and the binding and release of phenolic and flavonoid compounds to and from the food matrix during digestion (59). In general, the findings indicate that both TPC and TFC values decreased in both sample groups (GTK and GTK-P) as a result of gastrointestinal digestion. Similarly, Wanyo et al. (60) observed a decrease in the TPC and TFC of green and black mulberry leaf kombuchas following gastrointestinal digestion. By contrast, Abuduaibifu and Tamer (61) observed an increase in the total phenolic content of black tea and kombuchas made from black and red goji berries after gastric and intestinal digestion.

**Table 6:** Results for TPC and TFC bioavailability for GTK and GTK-P samples.

Phases	Storage (Days)	TPC (mg GAE/L)		TFC (mg CE/L)	
		GTK	GTK-P	GTK	GTK-P
Undigested	Day 0	310.31 ± 1.66aA	337.05 ± 3.01aB	38.78 ± 0.70aA	46.13 ± 1.46aB
	Day 7	313.34 ± 1.51abA	341.20 ± 2.61abB	38.91 ± 0.70aA	44.95 ± 1.00aB
	Day 14	315.85 ± 1.03bcA	344.12 ± 2.60bcB	39.37 ± 1.03aA	46.38 ± 0.5aB
	Day 21	317.95 ± 1.82cA	348.49 ± 1.55cB	39.95 ± 0.72aA	45.51 ± 2.07aB
Oral digestion	Day 0	148.63 ± 2.16aA	167.99 ± 2.10aB	30.41 ± 1.01aA	36.81 ± 0.86aB
	Day 7	149.28 ± 2.17aA	168.73 ± 2.11aB	30.51 ± 1.01aA	36.93 ± 0.86aB
	Day 14	151.60 ± 2.21aA	171.35 ± 2.15aB	31.02 ± 1.03aA	37.55 ± 0.88aB
	Day 21	153.08 ± 2.23aA	172.84 ± 2.08aB	31.32 ± 1.04aA	37.35 ± 1.12aB
Gastric digestion	Day 0	109.41 ± 1.24aA	122.79 ± 4.51aB	16.76 ± 0.78aA	20.48 ± 1.52aB
	Day 7	109.90 ± 1.25aA	123.33 ± 4.53aB	16.81 ± 0.79aA	20.55 ± 1.53aB
	Day 14	111.60 ± 1.27aA	125.25 ± 4.60aB	17.09 ± 0.80aA	21.22 ± 1.37aB
	Day 21	112.51 ± 1.03aA	126.48 ± 4.65aB	16.93 ± 1.24aA	21.09 ± 1.58aB
Intestinal digestion	Day 0	75.99 ± 2.07aA	86.96 ± 0.58aB	6.68 ± 0.34aA	9.15 ± 0.77aB
	Day 7	76.32 ± 2.08aA	87.65 ± 1.11abB	7.36 ± 0.84aA	9.51 ± 0.93aB
	Day 14	77.51 ± 2.11aA	88.70 ± 0.59abB	6.92 ± 0.28aA	10.00 ± 0.41aB
	Day 21	78.27 ± 2.13aA	89.57 ± 0.60bB	6.88 ± 0.35aA	10.42 ± 1.16aB
Recovery %	Day 0	24.49 ± 0.80aA	25.80 ± 0.16aB	17.23 ± 1.19aA	19.83 ± 1.55aA
	Day 7	24.36 ± 0.58aA	25.69 ± 0.33aB	18.94 ± 2.36aA	21.16 ± 2.03aA
	Day 14	24.54 ± 0.74aA	25.78 ± 0.12aB	17.59 ± 1.17aA	21.55 ± 0.72aB
	Day 21	24.62 ± 0.65aA	25.70 ± 0.19aA	17.23 ± 1.18aA	22.92 ± 2.59aB

TPC, total phenolic compound; TFC, total flavonoid content; GAE, gallic acid equivalent; CE, catechin equivalent; GTK, Kombucha tea fermented with green tea; GTK-P, Kombucha tea fermented with green tea with optimized propolis addition. The results are presented as the mean ± standard deviation. Different lowercase letters (a–c) within the same column indicate statistically significant differences between storage days (*p* < 0.05). Different uppercase letters (A–B) within the same row indicate statistically significant differences between products on the same day (*p* < 0.05).

The phenolic recovery percentages calculated at the end of the digestion process provide insight into the bioavailability of phenolic compounds. Taking into account the effect of the digestion process on phenolic compounds, the highest recovery rate was found to be 25.80 ± 0.16% in the GTK-P (0 day) sample. The minimum recovery was found to be 24.36 ± 0.58% in the GTK (day 7) sample. Deǧirmencioǧlu et al. (62) found the bioavailability of kombucha tea fermented with green tea to be 56.72%, higher than in the present study. Total flavonoid content showed recovery rates ranging from 17.23% to 18.94% for GTK and from 19.83% to 22.92% for GTK-P. The greatest loss in flavonoid content at the end of intestinal digestion was observed in the 0-day sample for both the GTK and GTK-P samples. The best recovery was detected on day 7 for the GTK sample and on day 21 for the GTK-P sample. The TFC and TPC recovery % results for the GTK-P samples (at 0, 7, 14, and 21 days) were higher than the corresponding values for the GTK samples at all time points. A study by Çalişkan et al. (63) evaluated the bioavailability of total phenolic content in three kombucha samples: green tea, aronia and green tea-aronia blend. The highest bioavailability rate was found to be 48.5% in aronia kombucha, followed by 31.4% in green tea kombucha and 28.58% in green tea-aronia kombucha. In our current study, adding propolis to green tea was found to provide higher TPC recovery (Table 6). When considered in terms of nutrition and functional food development, these findings indicate that the inclusion of propolis in kombucha formulations not only increases the initial phenolic content but also improves the stability and potential bioactivity of phenolic compounds during the digestion process.

In both GTK and GTK-P samples, the differences observed in the recovery percentage (TPC and TFC) on the 0th, 7th, 14th and 21st days of storage were found to be statistically insignificant (*p* > 0.05). Tamer et al. (64) examined the bioavailability of kombucha enriched with various plant extracts (lime blossom, lemon balm, sage, echinacea, mint and cinnamon) in their study. They observed an increase in TPC bioavailability at the start of storage (day 0) for all samples except echinacea and cinnamon kombucha, which showed a decrease after nine days under storage conditions. The lowest recovery occurred in the control sample (green-black tea kombucha) at both the beginning and end of storage, while the highest recovery was recorded for the lemon balm-enriched kombucha tea. In our study, the GTK sample showed an increase in initial (day 0) TPC recovery after 21 days of storage, while the GTK-P sample showed a slight decrease.

### 3.6 Sensory evaluation

The sensory analysis data for the GTK and GTK-P samples, organized by storage day, are presented in Table 7. When the sensory analysis parameters were evaluated overall, the lowest score points were observed for the smell results, while the highest score points were associated with the sourness values. No significant changes were observed in color, smell, taste and general acceptability scores in either the GTK-P or GTK samples over different storage days. Similarly, it has been reported that changes in smell scores during the 30-day storage period of black tea kombucha containing varying amounts of purple basil were not statistically significant (44). Upon completion of fermentation, the GTK-P samples were found to have higher scores for smell and color. Similar outcomes were noted in a study conducted by Bacanak and Keyvan (19).. This study found that adding propolis to black tea kombucha resulted in higher color and smell scores than the control group. Furthermore, the study concluded that the addition of propolis had a positive effect on the sensory parameters of kombucha tea. Dos Santos et al. (65) examined the effect of kombucha fermentation on sensory parameters, whereby sucrose and different honeys were added to mate tea as a carbohydrate source. It was determined that the addition of honey caused statistically insignificant changes in taste sensory parameters. Similarly, in our study, no statistically significant differences were found in the taste scores obtained from samples with propolis added to green tea. This suggests that the panelists preferred the simpler flavor profile of standard kombucha.

**Table 7:** Sensory analysis results for GTK and GTK-P samples.

Sensory Evaluation	Storage (Days)	GTK	GTK-P
Color	Day 0	6.07 ± 1.03aA	6.67 ± 0.98aA
	Day 7	6.00 ± 1.20aA	6.53 ± 0.83aA
	Day 14	6.40 ± 1.12aA	6.53 ± 0.99aA
	Day 21	6.00 ± 1.00aA	6.33 ± 0.90aA
Smell	Day 0	4.87 ± 2.17aA	5.93 ± 1.58aA
	Day 7	5.27 ± 2.02aA	5.40 ± 1.40aA
	Day 14	5.67 ± 1.99aA	5.73 ± 1.58aA
	Day 21	5.53 ± 2.07aA	5.27 ± 1.39aA
Taste	Day 0	6.53 ± 1.46aA	6.00 ± 1.65aA
	Day 7	6.47 ± 1.46aA	5.47 ± 1.25aA
	Day 14	6.07 ± 1.58aA	5.40 ± 1.76aA
	Day 21	5.47 ± 1.64aA	5.07 ± 1.79aA
General acceptability	Day 0	6.07 ± 1.44aA	6.20 ± 1.42aA
	Day 7	6.47 ± 1.30aA	6.00 ± 0.93aA
	Day 14	6.20 ± 1.47aA	5.80 ± 1.21aA
	Day 21	5.73 ± 1.16aA	5.53 ± 1.60aA
Sourness	Day 0	6.13 ± 0.92aA	5.60 ± 1.18aA
	Day 7	6.53 ± 1.19aA	6.20 ± 1.52abA
	Day 14	6.87 ± 1.30aA	6.53 ± 1.68abA
	Day 21	6.47 ± 1.81aA	7.33 ± 1.72bA

GTK, Kombucha tea fermented with green tea; GTK-P, Kombucha tea fermented with green tea with optimized propolis addition. The results are presented as the mean ± standard deviation. Different lowercase letters (a–c) within the same column indicate statistically significant differences between storage days (*p* < 0.05). Different uppercase letters (A–B) within the same row indicate statistically significant differences between products on the same day (*p* < 0.05).

The sour taste of kombucha tea stems from the presence of acetic acid, whereas bitterness is related to polyphenol content and can be masked by increasing the level of sweetness (66–68). In our study, the GTK-P sample had a lower initial sourness score (day 0), but a higher score as the storage period progressed. However, this increase did not create a statistically meaningful difference in sourness between the GTK-P and GTK samples during the storage duration. However, the sourness score obtained for the GTK-P sample on day 21 of storage was found to be 23.6% higher than the day 0 score (*p* < 0.05). The increased sourness detected in GTK-P samples during storage may be related to ongoing microbial metabolism converting residual sugars into organic acids. The production of these acids lowers the pH of the substrate medium. This decrease in pH contributes to the sour taste of kombucha (69, 70). The critical organic acids involved in forming this sour taste include acetic acid, gluconic acid and citric acid (71).

## 4 Conclusion

This article provides a thorough review of the bioactive potential and *in vitro* bioavailability of green tea kombucha enriched with propolis, employing a structured experimental design based on response surface methodology. The results clearly demonstrate that enriching kombucha with propolis significantly increases levels of bioactive compounds (TPC, TFC, and DPPH). These results were observed consistently over different storage periods (0, 7, 14, and 21 days), demonstrating the effectiveness of propolis supplementation and the chemical stability of the product. *In vitro* gastrointestinal digestion analyses confirmed that GTK-P samples exhibited significantly higher recovery rates for phenolic and flavonoid compounds than GTK samples. This finding is particularly important as it highlights the dual benefit of propolis enrichment: increased bioactive concentration and improved digestive stability. The increased bioavailability of phenolic compounds suggests that the therapeutic potential of functional foods depends on both compound content and physiological accessibility. Furthermore, sensory evaluation revealed that GTK-P samples remained acceptable to consumers in terms of color, aroma, odor, and overall impression throughout the 21-day storage period.

Future research may also investigate using GTK-P as a distribution matrix for other functional compounds, or its potential application in dietary interventions aimed at preventing chronic diseases. Further *in vivo* and clinical studies are needed to validate the observed *in vitro* bioavailability results and to evaluate the long-term health effects of GTK-P administration in humans. Additionally, the structure of an individual's gut microbiota can significantly influence the bioavailability of bioactive compounds with therapeutic potential. Investigating probiotic viability and microbial dynamics during fermentation with the addition of propolis may provide valuable insights into safety and microbial synergy. Exploring different types of propolis (e.g., regional or botanical variations) and green tea varieties may also contribute to the development of kombucha formulations with enhanced functional and sensory properties.

## Data Availability

The raw data supporting the conclusions of this article will be made available by the authors, without undue reservation.

## References

[1] Soltani M Farshadfar M Shirvani H Yaghotipoor A. Evaluation of the antibacterial effect of a beneficial compound based on the probiotic kombucha and honey. Honeybee Sci J. (2021) 11. doi: 10.22092/hbsj.2021.125166

[2] Soares MG de Lima M Schmidt VCR. Technological aspects of kombucha, its applications and the symbiotic culture (SCOBY), and extraction of compounds of interest: a literature review. Trends Food Sci Technol. (2021) 110:539–50. doi: 10.1016/j.tifs.2021.02.017

[3] Malbaša RV Lončar ES Vitas JS Canadanović-Brunet JM. Influence of starter cultures on the antioxidant activity of kombucha beverage. Food Chem. (2011) 127:1727–31. doi: 10.1016/j.foodchem.2011.02.048

[4] Antolak H Piechota D Kucharska A. Kombucha tea—a double power of bioactive compounds from tea and symbiotic culture of bacteria and yeasts (SCOBY). Antioxidants. (2021) 10:1541. doi: 10.3390/antiox10101541 34679676 PMC8532973

[5] Leal J Suárez L Jayabalan R Oros J Escalante-Aburto A. A review on health benefits of kombucha nutritional compounds and metabolites. CyTA J Food. (2018) 16:390–9. doi: 10.1080/19476337.2017.1410499

[6] Jayabalan R Malbaša RV Lončar ES Vitas JS Sathishkumar M. A review on kombucha tea—microbiology, composition, fermentation, beneficial effects, toxicity, and tea fungus. Compr Rev Food Sci Food Saf. (2014) 13:538–50. doi: 10.1111/1541-4337.12073 33412713

[7] de Miranda JF Ruiz LF Silva CB Uekane TM Silva KA Gonzalez AGM . Kombucha: a review of substrates, regulations, composition, and biological properties. J Food Sci. (2022) 87:503–27. doi: 10.1111/1750-3841.16029 35029317

[8] Battikh H Chaieb K Bakhrouf A Ammar E. Antibacterial and antifungal activities of black and green kombucha teas. J Food Biochem. (2013) 37:231–6. doi: 10.1111/j.1745-4514.2011.00629.x

[9] Bhattacharya S Gachhui R Sil PC. Effect of kombucha, a fermented black tea in attenuating oxidative stress mediated tissue damage in alloxan induced diabetic rats. Food Chem Toxicol. (2013) 60:328–40. doi: 10.1016/j.fct.2013.07.051 23907022

[10] Aloulou A Hamden K Elloumi D Ali MB Hargafi K Jaouadi B . Hypoglycemic and antilipidemic properties of kombucha tea in alloxan-induced diabetic rats. BMC Complement Altern Med. (2012) 12:1–9. doi: 10.1186/1472-6882-12-63 22591682 PMC3403982

[11] Srihari T Arunkumar R Arunakaran J Satyanarayana U. Downregulation of signalling molecules involved in angiogenesis of prostate cancer cell line (PC-3) by kombucha (lyophilized). Biomed Prev Nutr. (2013) 3:53–8. doi: 10.1016/j.bionut.2012.08.001

[12] Wang Y Ji B Wu W Wang R Yang Z Zhang D . Hepatoprotective effects of kombucha tea: identification of functional strains and quantification of functional components. J Sci Food Agric. (2014) 94:265–72. doi: 10.1002/jsfa.6245 23716136

[13] De Souza ACF Rigobello ES Reitz FAC Marques LLM Pereira CDO Perdoncini MRFG. Antifungal potential of hibiscus tea and fermented kombucha. Chem Eng Trans. (2023) 102:187–92. doi: 10.3303/CET23102032

[14] Aspiyanto A Susilowati A Iskandar JM Melanie H Maryati Y Lotulung PD . Characteristic of fermented spinach (*Amaranthus* spp.) polyphenol by kombucha culture for antioxidant compound. AIP Conf Proc. AIP Publishing (2017) 1803:020018. doi: 10.1063/1.4973145

[15] Li S Zhang Y Gao J Li T Li H Mastroyannis A . Effect of fermentation time on physiochemical properties of kombucha produced from different teas and fruits: comparative study. J Food Qual. (2022) 2022:2342954. doi: 10.1155/2022/2342954

[16] Watawana MI Jayawardena N Gunawardhana CB Waisundara VY. Health, wellness, and safety aspects of the consumption of kombucha. J Chem. (2015) 2015:591869. doi: 10.1155/2015/591869

[17] Sinija V Mishra HN. Green tea: health benefits. J Nutr Environ Med. (2008) 17:232–42. doi: 10.1080/13590840802518785

[18] Reyes-Flores S Pereira TSS Ramírez-Rodrigues MM. Optimization of hempseed-added kombucha for increasing the antioxidant capacity, protein concentration, and total phenolic content. Beverages. (2023) 9:50. doi: 10.3390/beverages9020050

[19] Bacanak RT Keyvan E. Assessment of propolis-fermented kombucha tea's microbiological, physicochemical and sensory characteristics. Emirates J Food Agric. (2024) 36:1–7. doi: 10.3897/ejfa.2024.118977

[20] Zullkiflee N Taha H Usman A. Propolis: its role and efficacy in human health and diseases. Molecules. (2022) 27:6120. doi: 10.3390/molecules27186120 36144852 PMC9504311

[21] Marcucci MC. Propolis: chemical composition, biological properties and therapeutic activity. Apidologie. (1995) 26:83–99. doi: 10.1051/apido:19950202

[22] Anjum SI Ullah A Khan KA Attaullah M Khan H Ali H . Composition and functional properties of propolis (bee glue): a review. Saudi J Biol Sci. (2019) 26:1695–703. doi: 10.1016/j.sjbs.2018.08.013 31762646 PMC6864204

[23] Lotfy M. Biological activity of bee propolis in health and disease. Asian Pac J Cancer Prev. (2006) 7:22–31. 16629510

[24] Braakhuis A. Evidence on the health benefits of supplemental propolis. Nutrients. (2019) 11:2705. doi: 10.3390/nu11112705 31717277 PMC6893770

[25] Shahidi F Peng H. Bioaccessibility and bioavailability of phenolic compounds. J Food Bioactives. (2018) 4:11–68. doi: 10.31665/JFB.2018.4162

[26] Fairweather-Tait S Southon S. Bioavailability of nutrients. In:Caballero B, editor. Encyclopedia of Food Sciences and Nutrition (Second Edition). Academic Press (2003). p. 478–84. doi: 10.1016/B0-12-227055-X/00096-1

[27] Benito P Miller D. Iron absorption and bioavailability: an updated review. Nutr Res. (1998) 18:581–603. doi: 10.1016/S0271-5317(98)00044-X

[28] Ketnawa S Suwannachot J Ogawa Y. *In vitro* gastrointestinal digestion of crisphead lettuce: changes in bioactive compounds and antioxidant potential. Food Chem. (2020) 311:125885. doi: 10.1016/j.foodchem.2019.125885 31780224

[29] Etcheverry P Grusak MA Fleige LE. Application of *in vitro* bioaccessibility and bioavailability methods for calcium, carotenoids, folate, iron, magnesium, polyphenols, zinc, and vitamins B6, B12, D, and E. Front Physiol. (2012) 3:317. doi: 10.3389/fphys.2012.00317 22934067 PMC3429087

[30] Stinco CM Fernandez-Vazquez R Escudero-Gilete ML Heredia FJ Melendez-Martinez AJ Vicario IM. Effect of orange juice's processing on the color, particle size, and bioaccessibility of carotenoids. J Agric Food Chem. (2012) 60:1447–55. doi: 10.1021/jf2043949 22250727

[31] Cardoso RR Neto RO Dos Santos D'Almeida CT do Nascimento TP Pressete CG Azevedo L . Kombuchas from green and black teas have different phenolic profile, which impacts their antioxidant capacities, antibacterial and antiproliferative activities. Food Res Int. (2020) 128:108782. doi: 10.1016/j.foodres.2019.108782 31955755

[32] Singleton VL Rossi JA. Colorimetry of total phenolics with phosphomolybdic-phosphotungstic acid reagents. Am J Enol Vitic. (1965) 16:144–58. doi: 10.5344/ajev.1965.16.3.144

[33] Zhishen J Mengcheng T Jianming W. The determination of flavonoid contents in mulberry and their scavenging effects on superoxide radicals. Food Chem. (1999) 64:555–9. doi: 10.1016/S0308-8146(98)00102-2

[34] Brand-Williams W Cuvelier M-E Berset C. Use of a free radical method to evaluate antioxidant activity. LWT-Food Sci Technol. (1995) 28:25–30. doi: 10.1016/S0023-6438(95)80008-5

[35] Portu J López R Santamaría P Garde-Cerdán T. Elicitation with methyl jasmonate supported by precursor feeding with phenylalanine: effect on Garnacha grape phenolic content. Food Chem. (2017) 237:416–22. doi: 10.1016/j.foodchem.2017.05.126 28764015

[36] Sezer B Apaydin H Bilge G Boyaci IH. Detection of *Pistacia vera* adulteration by using laser induced breakdown spectroscopy. J Sci Food Agric. (2019) 99:2236–42. doi: 10.1002/jsfa.9418 30324635

[37] Minekus M Alminger M Alvito P Ballance S Bohn T Bourlieu C . A standardised static in vitro digestion method suitable for food–an international consensus. Food Funct. (2014) 5:1113–24. doi: 10.1039/C3FO60702J 24803111

[38] Yıkmış S Bozgeyik E Simşek MA. Ultrasound processing of verjuice (unripe grape juice) vinegar: effect on bioactive compounds, sensory properties, microbiological quality and anticarcinogenic activity. J Food Sci Technol. (2020) 57:3445–56. doi: 10.1007/s13197-020-04379-5 32728291 PMC7374649

[39] Ögüt S Türkol M Yikmiş S Bozgeyik E Abdi G Kocyigit E . Ultrasound-assisted enhancement of bioactive compounds in hawthorn vinegar: a functional approach to anticancer and antidiabetic effects. Ultrason Sonochem. (2025):107245. doi: 10.1016/j.ultsonch.2025.107245 39879805 PMC11814703

[40] Jakubczyk K Kałduńska J Kochman J Janda K. Chemical profile and antioxidant activity of the kombucha beverage derived from white, green, black and red tea. Antioxidants. (2020) 9:447. doi: 10.3390/antiox9050447 32455926 PMC7278673

[41] Fraiz GM Bonifácio DB Lacerda UV Cardoso RR Corich V Giacomini A . Green tea kombucha impacts inflammation and salivary microbiota in individuals with excess body weight: a randomized controlled trial. Nutrients. (2024) 16:3186. doi: 10.3390/nu16183186 39339787 PMC11435194

[42] Lopes GA Fidelis PC Almeida BMd Almeida JJ Ientz GdAS Binda NS . Antioxidant activity, sensory analysis and acceptability of red fruit juice supplemented with Brazilian green propolis. Food Sci Technol. (2021) 42:e13521. doi: 10.1590/fst.13521

[43] Silva KA Uekane TM Miranda JFd Ruiz LF Motta JCBd Silva CB . Kombucha beverage from non-conventional edible plant infusion and green tea: characterization, toxicity, antioxidant activities and antimicrobial properties. Biocatal Agric Biotechnol. (2021) 34:102032. doi: 10.1016/j.bcab.2021.102032

[44] Yikmiş S Tuggüm S. Evaluation of microbiological, physicochemical and sensorial properties of purple basil kombucha beverage. Turk J Agric Food Sci Technol. (2019) 7:1321–7. doi: 10.24925/turjaf.v7i9.1321-1327.2550

[45] Pant K Sharma A Chopra HK Nanda V. Impact of biodiversification on propolis composition, functionality, and application in foods as natural preservative: a review. Food Control. (2024) 155:110097. doi: 10.1016/j.foodcont.2023.110097

[46] Fidan M Inal B Tokgün O Çelikkaya B Tegin I Yabalak E. Propolis as a functional plant-derived food: antioxidant and anti-cancer properties from Sirnak and Hakkari regions. Eur Food Res Technol. (2025) 251:1–14. doi: 10.1007/s00217-025-04806-x

[47] Birinci C. Kolayli S. A comparative study of phenolic and antioxidant properties of propolis and sumac (*Rhus coriaria* L.). Uludag Bee J. (2025) 25:131–9. doi: 10.31467/uluaricilik.1624649

[48] Nikam K Bhusari S Wakte P. High performance liquid chromatography method validation and forced degradation studies of chrysin. J Res Pharm. (2023) 27:264–75. doi: 10.29228/jrp.309

[49] Panchal NS Lodha SR Sen D Khanra S Shah NR Sindhi FA . Unlocking the potential of natural products: a comprehensive review of chrysin derivatives and their biological activities. Arab J Chem. (2026) 19:4292025. doi: 10.25259/AJC_429_2025

[50] Engelmann MD Hutcheson R Cheng IF. Stability of ferric complexes with 3-hydroxyflavone (flavonol), 5,7-dihydroxyflavone (chrysin), and 3',4'-dihydroxyflavone. J Agric Food Chem. (2005) 53:2953–60. doi: 10.1021/jf048298q 15826045

[51] Gantner M Piotrowska A Kostyra E Hallmann E Ponder A Sionek B . Influence of herbal additives on the physicochemical, microbiological, polyphenolic, and sensory profile of green tea-based kombucha. Foods. (2025) 14:3497. doi: 10.3390/foods14203497 41154033 PMC12563013

[52] Pobiega K Kraśniewska K Gniewosz M. Application of propolis in antimicrobial and antioxidative protection of food quality–a review. Trends Food Sci Technol. (2019) 83:53–62. doi: 10.1016/j.tifs.2018.11.007

[53] Everette JD Bryant QM Green AM Abbey YA Wangila GW Walker RB. Thorough study of reactivity of various compound classes toward the Folin– Ciocalteu reagent. J Agric Food Chem. (2010) 58:8139–44. doi: 10.1021/jf1005935 20583841 PMC4075968

[54] Tomás-Barberán FA Espín JC. Phenolic compounds and related enzymes as determinants of quality in fruits and vegetables. J Sci Food Agric. (2001) 81:853–76. doi: 10.1002/jsfa.885

[55] Bauer-Petrovska B Petrushevska-Tozi L. Mineral and water soluble vitamin content in the kombucha drink. Int J Food Sci Technol. (2000) 35:201–5. doi: 10.1046/j.1365-2621.2000.00342.x

[56] Kluz MI Pietrzyk K Pastuszczak M Kacaniova M Kita A Kapusta I . Microbiological and physicochemical composition of various types of homemade kombucha beverages using alternative kinds of sugars. Foods. (2022) 11:1523. doi: 10.3390/foods11101523 35627093 PMC9141729

[57] Rendueles E Mauriz E Sanz-Gómez J González-Paramás AM Vallejo-Pascual ME Adanero-Jorge F . Biochemical profile and antioxidant properties of propolis from northern Spain. Foods. (2023) 12:4337. doi: 10.3390/foods12234337 38231851 PMC10706490

[58] Izol E Turhan M. In-depth phytochemical profile by LC-MS/MS, mineral content by ICP-MS, and *in-vitro* antioxidant, antidiabetic, antiepilepsy, anticholinergic, and antiglaucoma properties of Bitlis propolis. Life. (2024) 14:1389. doi: 10.3390/life14111389 39598187 PMC11596023

[59] Hollman PC van Trijp JM Buysman MN van der Gaag MS Mengelers MJ de Vries JH . Relative bioavailability of the antioxidant flavonoid quercetin from various foods in man. FEBS Lett. (1997) 418:152–6. doi: 10.1016/S0014-5793(97)01367-7 9414116

[60] Wanyo P Chamsai T Toontom N Nghiep LK Tudpor K. Evaluation of *in vitro* digested mulberry leaf tea kombucha: a functional fermented beverage with antioxidant, anti-inflammatory, antihyperglycemic, and antihypertensive potentials. Fermentation. (2025) 11:258. doi: 10.3390/fermentation11050258

[61] Abuduaibifu A Tamer CE. Evaluation of physicochemical and bioaccessibility properties of goji berry kombucha. J Food Process Preserv. (2019) 43:e14077. doi: 10.1111/jfpp.14077

[62] Deǧirmencioǧlu N Yildiz E Sahan Y Güldas M Gürbüz O. Impact of tea leaves types on antioxidant properties and bioaccessibility of kombucha. J Food Sci Technol. (2021) 58:2304–12. doi: 10.1007/s13197-020-04741-7 33967327 PMC8076432

[63] Çalişkan Z Yildiz E Güldaş M Gürbüz O. Bioactive and anti-carcinogenic properties of kombucha prepared with *Aronia melanocarpa* juice. Yeni Yüzyil J Med Sci. (2023) 4:198–206. doi: 10.46629/JMS.2023.137

[64] Tamer CE Suna S Karabacak AO Ozcan T Ersan LY Kaya BT . Evaluation of bioaccessibility and functional properties of kombucha beverages fortified with different medicinal plant extracts. Turk J Agric For. (2021) 45:13–32. doi: 10.3906/tar-2003-75

[65] Santos DFd Leonarski E Rossoni MA Alves V Francisco CTdP Pinto VZ . Honey-kombucha beverage with yerba maté infusion: development, polyphenols profile, and sensory acceptance. Int J Gastron Food Sci. (2024) 36:100909. doi: 10.1016/j.ijgfs.2024.100909

[66] Kitwetcharoen H Phung LT Klanrit P Thanonkeo P Thanonkeo S Tippayawat P . Kombucha healthy drink—recent advances in production, chemical composition and health benefits. Fermentation. (2023) 9:48. doi: 10.3390/fermentation9010048

[67] Bishop P Pitts ER Budner D Thompson-Witrick KA. Kombucha: biochemical and microbiological impacts on the chemical and flavor profile. Food Chem Adv. (2022) 1:100025. doi: 10.1016/j.focha.2022.100025

[68] Jayabalan R Marimuthu S Swaminathan K. Changes in content of organic acids and tea polyphenols during kombucha tea fermentation. Food Chem. (2007) 102:392–8. doi: 10.1016/j.foodchem.2006.05.032

[69] Essiedu JA Areerate P Withayagiat U. Evaluation of physiochemical composition, phenolic compounds, and antioxidant activity of kombucha produced from *Thunbergia laurifolia* as a potential functional food. Int J Food Sci Technol. (2024) 59:6999–7010. doi: 10.1111/ijfs.17408

[70] Onsun B Toprak K Sanlier N. Kombucha tea: a functional beverage and all its aspects. Curr Nutr Rep. (2025) 14:69. doi: 10.1007/s13668-025-00658-9 40411688 PMC12103323

[71] Tran T Grandvalet C Verdier F Martin A Alexandre H Tourdot-Maréchal R. Microbiological and technological parameters impacting the chemical composition and sensory quality of kombucha. Compr Rev Food Sci Food Saf. (2020) 19:2050–70. doi: 10.1111/1541-4337.12574 33337078

